# Supplementary material for: eNEMAL, an enhancer RNA transcribed from a distal MALAT1 enhancer, promotes NEAT1 long isoform expression
Source: PLoS One. 2021 May 21;16(5):e0251515. doi: 10.1371/journal.pone.0251515 (PMC8139514; doi:10.1371/journal.pone.0251515)
Supplement: S1 Fig — The final PCR products were loaded in the agarose gel, and the band indicated as #1 and #2 were cut for sequencing. The sequences identified from Sanger sequencing as well as the oligo(dT)-adaptor primer used for cDNA synthesis were shown. (PDF) [file pone.0251515.s001.pdf]

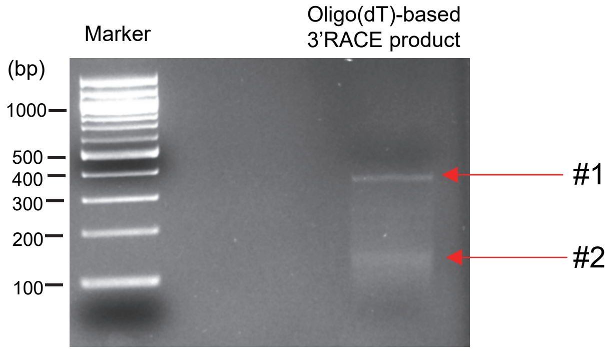

Sequence from band #1 – eNEMAL, partial (location: 11q13.1)

5'- TTCTCGCCCTTCATCCTCTGCTCCCTCACTTAGGCTCCAATTCCTCCTGATTTTGTTCTGTTTTTCCCTTCGGGCATCCAAGGCAGCC
ACCTGCCCAGGCCTGGGCCTGTTCAAACACTGTCTCCGCTGTACTCCCTAGCTCCTTGAACCCCTACATTGTCTGCATTCAGGAAGTT
TTGTGGCTTTTTAGGATTTTTTTTAAACACAGGGTCTGGCTCTGTTGCCCAGGCCGGAGTGCAGTAGTGTGATCATAGCTCACTGTAA
CCTCGAACTCCTGGGCTCCAGCCATAGGAAGCTTTTAATAAAACAACCTTTGCAAAAAAAAAAAAAACGCGTGCGCGTTTAAACGC -3'

Poly(A) primed by oligo(dT) Sequence complement to Adaptor

Sequence of band #2 – CTSD, partial (Cathepsin D; location: 11p15.5)

5'- TTCTCGCCCTTCATCCTCTGCACCTGACCTCTGTTGTCCTCCCCTTGGGCGGCTGAGAGCCCCAGCTGACATGGAAATACAGTTGTTG -3'
GCCTCCGGCCTCCCCCCAAAAAAAAAAAAAACGCGTGCGCGTTTAAACGC

Poly(A) primed by oligo(dT) Sequence complement to Adaptor

Oligo(dT)-adaptor primer used for cDNA synthesis

5' GCTCGCGAGCGCGTTTAAACGCGCACGCGTTTTTTTTTTTTTTTTTTVN 3'

Target sequence of the primer used for RACE 2nd PCR Oligo(dT)

S1 Fig. Sequences identified from Oligo(dT)-based conventional 3' RACE.
The final PCR products were loaded in the agarose gel, and the band indicated as #1 and #2 were cut for sequencing. The sequences identified from Sanger sequencing as well as the oligo(dT)-adaptor primer used for cDNA synthesis were shown.
