# Supplementary material for: eNEMAL, an enhancer RNA transcribed from a distal MALAT1 enhancer, promotes NEAT1 long isoform expression
Source: PLoS One. 2021 May 21;16(5):e0251515. doi: 10.1371/journal.pone.0251515 (PMC8139514; doi:10.1371/journal.pone.0251515)
Supplement: S2 Fig — Products from the final PCR were loaded in the agarose gel, and the band indicated with the red arrow was cut for sequencing. The sequences identified from Sanger sequencing were shown. The 3’ end of the transcript detected by 3’ RLM-RACE was located from 84 nt downstream from the cleavage site determined by conventional 3’ RACE (blue arrowhead). (PDF) [file pone.0251515.s002.pdf]

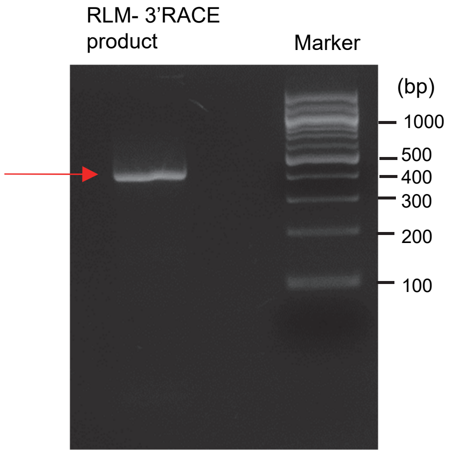


Sequence – eNEMAL, partial (location: 11q13.1)

TTCTCGCCCTTCATCCTCTGCTCCCTCACTTAGGCTCCAATTCCTCCTGATTTTGTTCTGTTTTTCCCTTCGGGCATCCAAGGCAGCCACC

TGCCCAGGCCTGGGCCTGTTCAAACACTGTCTCCGCTGTACTCCCTAGCTCCTTGAACCCCTACATTGTCTGCATTCAGGAAGTTTTGTGG

CTTTTTAGGATTTTTTTTAAACACAGGGTCTGGCTCTGTTGCCCAGGCCGGAGTGCAGTAGTGTGATCATAGCTCACTGTAACCTCGAACT

CCTGGGCTCCAGCCATAGGAAGCTTTTAATAAAACAACCTTTGCTCCGACCGCACCCTGGCCTTGCTGGATAAAGCCAGGCCTCTCTCTTG

GGTTACAGCAGGCCCTTCAGGACTTGGCTGCCCCTCTACGCGTGCGCGTTTAAACGC

additional 84 nt        Oligo adaptor ligated to RNA

**S2 Fig. Sequences identified from RNA ligase-mediated 3' RACE (3' RLM-RACE).** Products from the final PCR were loaded in the agarose gel, and the band indicated with the red arrow was cut for sequencing. The sequences identified from Sanger sequencing were shown. The 3' end of the transcript detected by 3' RLM-RACE was located from 84 nt downstream from the cleavage site determined by conventional 3' RACE (blue arrowhead).
