# Supplementary material for: eNEMAL, an enhancer RNA transcribed from a distal MALAT1 enhancer, promotes NEAT1 long isoform expression
Source: PLoS One. 2021 May 21;16(5):e0251515. doi: 10.1371/journal.pone.0251515 (PMC8139514; doi:10.1371/journal.pone.0251515)
Supplement: S3 Fig — Alignment generated as described in Fig 3. Accession numbers: Homo sapiens MT773342, Pan troglodytes NC_036890, Pan paniscus CM003394, Gorilla gorilla NC_044613, Pongo abelii NC_036914, Nomascus leucogenys NC_044384, Macaca fascicularis NC_022285, Macaca mulatta NC_041767, Macaca nemestrina KQ007745.1, Papio anubis NC_044989, Rhinopithecus bieti MCGX01000834.1, Rhinopithecus roxellana KN295605.1, Theropithecus gelada QGDE01000660.1, Colobus angolensis KN980607.1, Piliocolobus tephrosceles PDMG02000207.1, Chlorocebus sabaeus NC_044384, Mandrillus leucophaeus KN979072.1, Aotus nancymaae KZ200996.1, Callithrix jacchus NC_048393, Cebus capucinus imitator KV389528.1, and Saimiri boliviensis JH378199.1. (PDF) [file pone.0251515.s003.pdf]

```
                                        10        20        30        40        50        60        70        80        90       100
                              ....|....|....|....|....|....|....|....|....|....|....|....|....|....|....|....|....|....|....|....|
Homo_sapiens                  GCAGGGGCCGCTGGGAGTCCCGCGCTGCCTCCCC---TGC-CCCC---------GCGCCGGGG----------CCACGT-CCCCCA----------GCCA
Pan_troglodytes               GCAGGGGCCGCTGGGAGTCCCGCGCTGCCTCCCC---TGC-CCCC---------GCGCCGGGG----------CCACCT-CCCCCA----------GCCA
Pan_paniscus                  GCAGGGGCCGCTGGGAGTCCCGCGCTGCCTCCCC---TGC-CCCC---------GCGCCGGGG----------CCACTT-CCCCCA----------GCCA
Gorilla_gorilla               GCAGGGGCCGCTGGGAGTCCCGCGCTGCCTCCCC---TGC-CCCC---------GCGCCGGGG----------CCACCT-CCCCCA----------GCCA
Pongo_abelii                  GCAGGGGCCGCTGGGAGTCCCGCGCTGCCTCCCC---TGC-CCCC---------CCGCCGGGG----------CCACCT-CCCCCA----------GCCA
Nomascus_leucogenys           GCATGAGCCGCTGGGAGTCCCGCGCTGCCTCCCC---TGC-CCCT---------GCGCCGGGG----------CCACCT-CCCCCA----------GCCA
Macaca_nemestrina             GTGACGGCGGGTGGGCGTTCCGCGCTGCCTCCCC---TGT-CCCC---------GCGCCGGGG----------CCGCCT-CCTCCA----------GCCA
Macaca_fascicularis           GTGACGGCGGGTGGGCGTTCCGCGCTGCCTCCCC---TGC-CCCC---------GCGCCGGGG----------CCGCCT-TCTCCA----------GCCA
Macaca_mulatta                GTGACGGCGGGTGGGCGTTCCGCGCTGCCTCCCC---TGC-CCCC---------GCGCCGGGG----------CCGCCT-CCTCCA----------GCCA
Papio_anubis                  GTGACGGCGGGTGGGCGTTCCGCGCTGCCTCCCC---TGC-CCCC---------GCGCCGGGG----------CCGCTT-CCTCCA----------GCCA
Rhinopithecus_roxellana       GTGACGGCGGGTGGGCGTTCCGCGCTGCCTCCCC---TGC-CCCC---------GCGCCGGGG----------CCGCCT-CCTCCA----------GCCA
Theropithecus_gelada          GTGACGGCGGGTGGGCGTTCCGCGCTGCCTCCCC---TGC-CCCC---------GCGCCGGGG----------CCGCCT-CCTCCA----------GCCA
Colobus_angolensis            GTGACGGCGGGTGGGCGTTCCGCGCTGCCTCCCC---TGC-CCCC---------GCGCCGGGG----------CCGCCT-CCTCCA----------GCCA
Rhinopithecus_bieti           GTGACGGCGGGTGGGCGTTCCGCGCTGCCTCCCC---TGC-CCCC---------GCGCCGGGG----------CCGCCT-CCTCCA----------GCCA
Piliocolobus_tephrosceles     GTGACGGCGGGTGGGCGTTCCGCGCTGCCTCCCC---TGC-CCCC---------GCGCCGGGG----------CCGCCT-CCTCCA--------------
Chlorocebus_sabaeus           GTGACGGCGGGTGGGCGTTCCGCGCTGCCTCCCCCGCTGCTCCCC---------GCGCCGGCCAGGTCCCCTC-CGCCT-CCTCCA----------GCCA
Mandrillus_leucophaeus        NNNNNNNNNNNNNNNNNNNNNNNNNNNNNNNNNNNNNNNNNNNNNNNNNNNNNNNNNNNNNNNNNNNNNNNNNNNNNNNNNNNNNNNNNNNNNNNNNNNN
Aotus_nancymaae               GCAGGGGCCGCTGGGAG------------TCCCC---TGCTCCCCCGCTGCTCCCCGCGCCGGG---------TCGCCTTCCTCCA-----------CCA
Callithrix_jacchus            GCAGGGGCCGCTGGGAGTCCCGCTCTGCCTGCGC---TGC-CCGCGCTGCCCCCGCTGCCCCC-GCTGCCCCCGCGCCGGCCGCCTTCCTAGCCAAGCCA
Cebus_capucinus_imitator      GCAGGGGCCGCTGGGAGTTCCGCGCTACTTTCGCTGCTTCCGCGGCCCCCCCGCCGCGCCGTGG---------CCGCCTTCCTCC---------CGGCCA
Saimiri_boliviensis           GCAGGGGCTGCTGGGAGTCCCGCGCTGCTTCCGC---TGCNNNNNNNNNNNNNNNNNNNNNNNNNNNNNNNNNNNNNNNNNNNNNNNNNNNNNNNNNNNN

                                       110       120       130       140       150       160       170       180       190       200
                              ....|....|....|....|....|....|....|....|....|....|....|....|....|....|....|....|....|....|....|....|
Homo_sapiens                  GGTCCCTTC-TTCCTGCCGTGTCCCCAGGGCAGGACCGCATCGGAGAGAGTCCGGGAGGAGGCCGCGCTCCTGGGGG-CTGGAGACCCGGCGGCGGTGGC
Pan_troglodytes               GGTCCCTTC-TTCCTGCCGTGTCCCCAGGGCAGGACCGCATCGGAGAGAGTCCGGGAGGAGGCCGCGCTCCTGGGGG-CTGGAGACCCGGCGGCGGTGGC
Pan_paniscus                  GGTCCCTTC-TTCCTGCCGTGTCCCCAGGGCAGGACCGCATCGGAGAGAGTCCGGGAGGAGGCCGCGCTCCTGGGGG-CTGGAGACCCGGCGGCGGTGGC
Gorilla_gorilla               GGTCCCTTC-TTCCTGCCGTGTCCCCAGGGCAGGACCGCATCGGAGAGAGTCCGGGAGGAGGCCGCGCTCCTGGGGG-CTGGAGACCCGGCGGCGGTGGC
Pongo_abelii                  GGTCCCCTC-TTCCTGCCGTGTCCCCAGGGCAGGACCGCATCGGAGAGAGTCCGGGAGGAGGCCGCGCTCCTGGGGG-CTGGAGACCCGGCGGCGGTGGC
Nomascus_leucogenys           GGTCCCCTC-TCCCTGCCGTGTCCCCAGGGCAGGACCGCATCGGAGAGAGTCCAGGAGGAGGCCGCGCTCCTGGGGG-CTGGAGACCCGGCGGCGGTGGC
Macaca_nemestrina             GGTCCCCCC-TCCCTGCCGTGTCCCCAGGCCGGGACCGCATTGGAGAGAGTCGGGGAGGAGGCCGCGCTCCCGGGGG-CTGGAGACCCGGCGGCGGTGAC
Macaca_fascicularis           GGTCCCCCC-TCCCTGCCGTGTCCCCAGGCCGGGACCGCATTGGAGAGAGTCGGGGAGGAGGCCGCGCTCCCGGGGG-CTGGAGACCCGGCGGCGGTGAC
Macaca_mulatta                GGTCCCCCC-TCCCTGCCGTGTCCCCAGGCCGGGACCGCATTGGAGAGAGTCGGGGAGGAGGCCGCGCTCCCGGGGG-CTGGGGACCCGGCGGCGGTGAC
Papio_anubis                  GGTCCCCCC-TCCCTGCCGTGTCCCCAGGCCGGGACCGCATTGGAGAGAGTCGGGGAGGAGGCTGCGCTCCCGGGGGGCTGGAGACCCGGCGGCGGTGAC
Rhinopithecus_roxellana       GGTCCCCTC-TCCCTGCCGTGTCCCCAGGCCGGGACCGCATTGGAGAGAGTCGGGGAGGAGGCCGCGCCCCCGGGGG-CTGGAGACCCGGCGGCGGTGGC
Theropithecus_gelada          GGTCCCCCCCTCCCTGCCGTGTCCCCAGGCCGGGACCGCATTGGAGAGAGTCGGGGAGGAGGCTGCGCTCCCGGGGG-CTGGAGACCCGGCGGCGGTGAC
Colobus_angolensis            GGTCCCCTC-TCCCTGCCGTGTCCCCAGGCCGGGACCGCATTGGAGAGAGTCGGTGAGGAGGCCGCGCTCCCGAGGG-CTGGAGACCCGGCGGCGGTGGC
Rhinopithecus_bieti           GGTCCCCTC-TCCCTGCCGTGTCCCCAGGCCGGGACCGCATTGGAGAGAGTCGGGGAGGAGGCCGCGCCCCCGGGG--CTGGAGACCCGGCGGCGGTGGC
Piliocolobus_tephrosceles     ----------TCCCTGCCGTGTCCCCAGGCCGGGACCGCATAGGAGAG--TCGGGGAGGAGGCCGCGCTCCCGAGGG-CTGGAGACCCGGCGGCGGTGGC
Chlorocebus_sabaeus           GGTCCCCTC-TCCCTGTCGTGTCCCCAGGCCGGGACCACATTGGCGAGAGTCGGGGAGGAGGCCGCGCTCCCGGGGG-CTGGAGACCCGGCGGCGGTGAC
Mandrillus_leucophaeus        NNNNNNNNNNNNNNNNNNNNNNNNNNNNNNNNNNNNCGCATTGGAGAGAGTCGGGGAGGAGGCCGCGCTCCCGGGGG-CTGGAGACCCGGCGGCGGTGAC
Aotus_nancymaae               GGTCCCCTC-TCCCCTCCGTGTCCCCAGGGTGGGACTCTGTCGGAGAGGGTGGGGGAGGAGGCCTCGTGCACGGGG--CTGGGGACCCCTCGGCGAGGGC
Callithrix_jacchus            GGTCCCCTC-TCCCCGCCGTGTCCCCAGCCCGGGACTGCGTCGGAGAGGGTGGGGGAGGAGGCCGCGTGCACGGGG--CCGGAGACCCCGCGGCGGTGGC
Cebus_capucinus_imitator      GGTCAGCTC-TCCCCGCTGTGTCCCCAGCCCGGGACTGCGCCGGAGAGGGTGGAGGAGGAGGCCGCGTGCACGGGG--CTGGAGACCCCGCGGCCGTGGC
Saimiri_boliviensis           NNNNNNNNNNNNNNNNNNNNNNNNNNNNNNNNNNNNTGCGTCGCAGAGGGTGGGGGAGGAGGCCGCGTGCACGGGG--CTGGAGTCCCCGCGGCGGTGGC
```

```
                                      210       220       230       240       250       260       270       280       290       300
                                ....|....|....|....|....|....|....|....|....|....|....|....|....|....|....|....|....|....|....|....|
Homo_sapiens                    CACTGCGCGCTGTTCCAGCCTTGAGG------GAGGGAACCGCGGGCC-TGGGTCCGCCTTGGGATTTGGGAACCGGTTTCCTGCCGGGTTGACCTAACT
Pan_troglodytes                 CACTGCGCGCTGTTCCAGCTTTGAGG------GAGGGAACCGCGGGCC-TGGGTCCGCCTTGGGATTTGGGAACCGGTTTCCTGCCGGGTTGACCTAACT
Pan_paniscus                    CACTGCGCGCTGTTCCAGCTTTGAGG------GAGGGAACCGCGGGCC-TGGGTCCGCCTTGGGATTTGGGAACCGGTTTCCTGCCGGGTTGACCTAACT
Gorilla_gorilla                 CACTGCGCGCTGTTCCAGCCTTGAGG------GAGGGAACCGCGGGCC-TGGGTCCGCCTTGGGATTTGGGAACCGGTTTCCTGCCGGGTTGACCTAACT
Pongo_abelii                    CACTGCGCGCTGTTCCAGCCTTGAGG------GAGGGAACCGCGGGCC-TGGGTCCGCCTTGGGATTTGGGAACCAGTTTCCTGCCGAGTTGACCTAACT
Nomascus_leucogenys             CACCGCGCGCTGTTCCAGCCTTGAGG------GAGGAAACCGCGGGCC-TGGGTCCGCCTTAGGATTTGGGAACCGGTTTCCTGCCGAGTTGACCTAACT
Macaca_nemestrina               CAAGGCGCGCTGTTCCAGCCTTGAGG------GAGGGAACCGCGGGCC-TGGGTCCGCCTTGGGATTTGGGAACCGGTTTCCTGCCAAGTTGACTTAACT
Macaca_fascicularis             CACGGCGCGCTGTTCCAGCCTTGAGG------GAGGGAACCGCGGGCC-TGGGTCCGCCTTGGGATTTGGGAACCGGTTTCCTGCCAAGTTGACTTAACT
Macaca_mulatta                  CACGGCGCGCTGTTCCAGCCTTGAGG------GAGGGAACCGCGGGCC-TGGGTCCGCCTTGGGATTTGGGAACCGGTTTCCTGCCAAGTTGACTTAACT
Papio_anubis                    CACGGCGCGCTCTTCCAGCCTTGAGG------GAGGGAACCGCGGGCC-TGGGTCCGCCTTGGGATTTGGGAACCGGTTTCCTGCCAAGTTGACTTAACT
Rhinopithecus_roxellana         CACGGCGCGCTGTTCCAGCCTTGAGG------GAGGGAACCGCGGGCC-TGGGTCCGCCTTGGGATTTGGGAACCGATATCCTGCCAAGTTGACTTAACT
Theropithecus_gelada            CACGGCGCGCTGTTCCAGCCTTGAGG------GAGGGAACCGCGGGCC-TGGGTCCGCCTTGGGATTTGGGAACCGGTTTCCTGCCAAGTTGACTTAACT
Colobus_angolensis              CACGGCGCGCTGTTCCAGCCTTGAGG------GAGAGAACCGCGGGCC-TGGGTCCGCCTTGGGATTTGGGAACCGATTTCCTGCCAAGTTGACTTAACT
Rhinopithecus_bieti             CACGGCGCGCTGTTCCAGCCTTGAGG------GAGGGAACCGCGGGCCCTGGGTCCGCCTTGGGATTTGGGAACCGATATCCTGCCAAGTTGACTTAACT
Piliocolobus_tephrosceles       CACGGCGCGCTGTTCCAGCCTTGAGGTTGAGGGAGGGAACCGCGGGCC-TGGGTCCGCCTTGGGATTTGGGAACCGATTTCCTGCCAAGTTGACTTAACT
Chlorocebus_sabaeus             CACGGCGCGCTGTTCCAGCCTTGAGG------GAGGGAACCGCGGGCC-TGGGTCCGCCTTGGGATTTGGGAACCGGTTTCCTGCCAAGTTGACTTAACT
Mandrillus_leucophaeus          CACGGCGCGCTGTTCCAGCCTTGAGG------GAGGGAACCGCGGGCC-TGGGTCCGCCTTGGGATTTGGGAACCGGTTTCCTGCCAAGTTGACTTAACT
Aotus_nancymaae                 CACTGTAGGCGGCTGCAGCCCTGAGC------GAGGGAACCGCGGGCC-TGGGTTGGCCTTGGGATTTGGGAACCGCTTTCCAGCCGAGTTGGCCTAACT
Callithrix_jacchus              CACTGCAGGCGGCTGCAGCCCTGAGG------GAGGGAACCGCGGGCC-TGGATTGGCGTTGGGATTTGGGAACCGCTTTCCAGCCCAGTTGGCCTAACT
Cebus_capucinus_imitator        CACTGCAGACGGCTGCAGCCCTGAGG------GAGGGAACCGCGGGCC-TGG----------GGATTTGGGAACCGCTTTCCAGCCGAGTTGGCCTAACT
Saimiri_boliviensis             CACTGCAGGCTGCTGCAGCCCTGAGG------GAGGGAACTGCGGGCC-TGGGTTGACCTTGGGATTTGGGAACCGCTTTCCTGTCGAGTTGGCATAACT

                                      310       320       330       340       350       360       370       380       390       400
                                ....|....|....|....|....|....|....|....|....|....|....|....|....|....|....|....|....|....|....|....|
Homo_sapiens                    TGAGCC-TTCAGTAGTTCCGGGGCGGGTGTTGTGGTCTCCGTTCCAAAGTCGGGAAAACGGTATCTGGGCTGGCGAAGGGCCGCGAGGGTGGTGGGAGGA
Pan_troglodytes                 TGAGCC-TTCAGTAGTTCCGGGGCGGGTGTTGTGGTCTCCGTTCCAAAGTCGGGAAAACGGTATCTGGGCTGGCGAAGGGCCGCGAGGGTGGTGGGAGGA
Pan_paniscus                    TGAGCC-TTCAGTAGTTCCGGGGCGGGTGTTGTGGTCTCCGTTCCAAAGTCGGGAAAACGGTATCTGGGCTGGCGAAGGGCCGCGAGGGTGGTGGGAGGA
Gorilla_gorilla                 TGAGCC-TTCAGTAGTTCCGGGGCGGGTGTTGTGGTCTCCGTTCCAAAGTCGGGAAAACGGTATCTGGGCTGGCGAAGGGCCGCGAGGGTGGTGGGAGGA
Pongo_abelii                    TGAGCC-TTCAGTAGTTCCGGGGCGGGTGTTGTGGTCTCCGTGCCAAAGTCGGGAAAACGGTATCTGGGCTGGCGAAGGGCCGCGAGGGTGGTGGGAGGA
Nomascus_leucogenys             TGAGCC-TTCAGTAGTTCCGGGGCGGGTGTTGTGGTCTCCGTTCCAAAGTCGGGAA---------------------GGGCCGCGAGGGTGGTGGGAGGA
Macaca_nemestrina               CGATCC-TTAAGTAGTTCCGGGGCGGGTGTTGTGGTCTCCGTTCCGAAGGCGGGAAAACGGTATCTGGGCTGGCGAAGGGCCGCGAGGGTGGTGGGAGGA
Macaca_fascicularis             CGATCC-TTAAGTAGTTCCGGGGCGGGTGTTGTGGTCTCCGTTCCGAAG-CGGGAAAACGGTATCTGGGCTGGCGAAGGGCCGCGAGGGTGGTGGGAGGA
Macaca_mulatta                  CGATCC-TTAAGTAGTTCCGGGGCGGGTGTTGTGGTCTCCGTTCCGAAG-CGGGAAAACGGTATCTGGGCTGGCGAAGGGCCGCGAGGGTGGTGGGAGGA
Papio_anubis                    CGATCC-TTAAGTAGTTCCGGGGCGGGTGTTGTGGTCTCCGTTCCGAAGGCGGGAAAACGGTATCTGGGCTGGCGAAGGGCCGCGAGGGTGGTGGGAGGA
Rhinopithecus_roxellana         TGATCC-TTCAGTAGTTCCGGGGCAGGTGTTGTGGTCTCCGTTCCAAAGGCGGGAAAACGGTATTTGGGCTGGCGAAGGGCCTTGAGGGTGGTGGGAGGA
Theropithecus_gelada            CGATCC-TTAAGTAGTTCCGGGGCGGGTGTTGTGGTCTCCGTTCCGAAGGCGGGAAAACGGTATCTGGGCTGGCGAAGGGCCGCGAGGGTGGTGGGAGGA
Colobus_angolensis              TGATCC-TTCAGTAGTTCCGGGGCAGGTGTTGTGGTCTCCCTTCCAAAGGCGGGAAAACGATATCTGGGCTGGCGAAGGGCCGCGAGGGTGGTGGGAGGA
Rhinopithecus_bieti             TGATCC-TTCAGTAGTTCCGGGGCAGGTGTTGTGGTCTCCGTTCCAAAGGCGGGAAAACGGTATCTGGGCTGGCGAAGGGCCTTGAGGGTGGTGGGAGGA
Piliocolobus_tephrosceles       TGATCC-TTCAGTAGTTCCGGGGCAGGTGTTGTGGTCTCCGTTCCAAAGGCGGGAAAACGATATCTGGGCTGGCGAAGGGCCGCGAGGGTGGTGGGAGGA
Chlorocebus_sabaeus             CGATCC-TTAAGTAGTTCCGGGGCGGGTGTTGTGGTCTCCGTTCCGAAGGCGGGAAAACGGTATCTGGGCTGGCGAAGGGCCGCGAGGGTGGTGGGAGGA
Mandrillus_leucophaeus          CGATCC-TTAAGTAGTTCCGGGGCAGGTGTTGTGGTCTCCGTTCCGAAGGCGGGAAAACGGTATCTGGGCTGGCGAAGGGCCGCGAGGGTGGT-------
Aotus_nancymaae                 TGATCC-TTCAGTAGTTCCTGCGCGGGTGTTGTGGTCTGCGTTCCAAAGGCGGGGAAACGGGATCTGGGCGGGCGCAGGGCCGCGAGGGTGGTGGGAGGA
Callithrix_jacchus              TGATCCCTTCAATAGTTCGTGCGCGGGTGTTGTGGTCTGCGTTCCAAAGGCGGGGAAACGGGATCTGGGCGGGCGCAGGGCTGCAAGG-TGGTGGGAGGA
Cebus_capucinus_imitator        TGATCC-TTCC--------TGCGCGGGTGTTGTGGTCTGCGTTCCAAAGGCGGGGAAACGGGATCTGGGCGGGCGCAGGGCCCCGAGGGTGGTGGGAGGA
Saimiri_boliviensis             TGATCC-TTCCGTGGTTCCTGCGCGGGTGTTGTGGTCTGCGTTCGAAAGGCGGGGAAACGGGATCTGGGCGGGCGCAGGGCCGCGAGGGTGGTGGGAGGA
```

```
                                      410       420       430       440       450       460       470       480       490       500
                             ....|....|....|....|....|....|....|....|....|....|....|....|....|....|....|....|....|....|....|....|
Homo_sapiens                 CCCTGTGGCTGCGCCGAACAGACCGCGGGAACCCACACTGAGTGGGGTGGGACG-GGCTGCCACCCCGGCTTTGTCAGCCCCATGCCCCCTTCTCGCCCT
Pan_troglodytes              CCCTGTGGCTGCGCCGAACAGACCGCGGGAACCCACACTGAGTGGGGTGGGACG-GGCTGCCACCCCGGCTTTGTCAGCCCCATGCCTCCTTCTCGCCCT
Pan_paniscus                 CCCTGTGGCTGCGCCGAACAGACCGCGGGAACCCACACTGAGTGGGGTGGGACG-GGCTGCCACCCCGGCTTTGTCAGCCCCATGCCTCCTTCTCGCCCT
Gorilla_gorilla              CCCTGTGGCTGCGCCGAACAGACCGCGGGAACCCACACTGAGTGGGGTGGGACG-GGCTGCCACCCCGGCTTTGTCAGCCCCATGCCCCCTTCTCGCCCT
Pongo_abelii                 CCCTGTGGCTGCGCCTACCAGACCGCGGGAACCCACACTGAGTGGGGTGGGACG-GGCTGCCACCCCGGCTTTGTCAGCCCCATGCCCCCTTCTCGCCCT
Nomascus_leucogenys          CCCTGTGGCTGCGCCGACCAGACCGCGGGAACCCACACTGAGTGGGGTGGGGCG-GGCTGCCACCCCGGCTTTGTCAGCCCCATGCTCCCTTCTGTCCCT
Macaca_nemestrina            CCCTGTGGCTGCGCCGACCAGACCGCGGGGACCCACACTGAGTGAGGAGGGGCG-GGCTGCCACCCCGGCTTTGTCAGCCCCATGCTCCCTTCT--CCCT
Macaca_fascicularis          CCCTGTGGCTGCGCCGACCAGACCGCGGGGACCCACACTGAGTGAGGAGGGGCG-GGCTGCCACCCCGGCTTTGTCAGCCCCATGCTCCCTTCT--CCCT
Macaca_mulatta               CCCTGTGGCTGCGCCGACCAGACCGCGGGGACCCACACTGAGTGAGGAGGGGCG-GGCTGCCACCCCGGCTTTGTCAGCCCCATGCTCCCTTCT--CCCT
Papio_anubis                 CCCTGTGGCTGCGCCGACCAGACCGCGGGGACCCACACTGAGTGAGGAGGGGCG-GGCTGCCACCCCGGCTTTGTCAGCCCCATGCTCCCTTCT--CCCT
Rhinopithecus_roxellana      CCCTGTGGCTGCGCCGACCAGACCGCGGGGACCCACACTGAGTGAGGTGGGGAG-GGCTGCCACCCCGGCTTTGTCAGCCCCGTGCTCCCTTCT--CCCT
Theropithecus_gelada         CCCTGTGGCTGCGCCGACCAGACCGCGGGGACCCACACTGAGTGAGGAGGGGCG-GGCTGCCACCCCGGCTTTGTCAGCCCCATGCTCCCTTCT--CCCT
Colobus_angolensis           CCCTGTAGCTGCGCCGACCAGACCGCGGGGACCCACACTGAGTGAGGTGGGGCG-GGCTGCCACCCCGGCTTTGTCAGCCCCGTGCTCTCTTCT--CCCT
Rhinopithecus_bieti          CCCTGTGGCTGCGCCGACCAGACCGCGGGGACCCACACTGAGTGAGGTGGGGAG-GGCTGCCACCCCGGCTTTGTCAGCCCCGTGCTCCCTTCT--CCCT
Piliocolobus_tephrosceles    CCCTGTGGCTGCGCCGACCAGACCGCGGGGACCCACAGTGAGTGAGGTGGGGCG-GGCTGCCACCCCCGCTTTGTCAGCCCCGTGCTCCCTTCT--CCCT
Chlorocebus_sabaeus          CCCTGTGGCTGCGCCGACCAGACCGCGGGGACCCACACTGAGTGAGGAGGGGCG-GGCTGCCACCCCGGCTTTGTCAGCCCCATGCTCCCTTCT--CCCG
Mandrillus_leucophaeus       -------------------------GGGGGGACCCCACTGAGTGAGGAGGGGCG-GGCTGCCACCCCGGCTTTGTCAGCCCCATGCTTCCTTCT--CCCT
Aotus_nancymaae              CCCTGTAGCTGCGCCGACCCGACCGTGGGGACCCACACGGAGTGGGATGGGGCGAGGCTGCCACCCCGGCTTTGTCAGCACGATGCTCCCTTCTGGACCT
Callithrix_jacchus           CC------------------GACCGTGGAGACCCACACGGAGTTGGGTGGGGCGAGGCTGCCACCCCGACTTTGTCAGCCCGATGCTCCCTTCTGGACCT
Cebus_capucinus_imitator     CCCTGTGGCTGCACCAACCCGACCGTGGGGACCCACAAGGAGTGGGATGGGGCGAGGCTGCCACCCCAGCTTTGTCAGCCCGATGCTCCCTTCTGGACCT
Saimiri_boliviensis          CCTTGTGGCTGCGCAGACCCGACCGTGGGGACCCACACGGAGTGGGGTGGGGCGAGGCTGCCACCCTGGCTTTGTCAGCCCGATGCTCCCTTCTGGACCT
```

```
                                      510       520       530       540       550       560       570       580       590       600
                             ....|....|....|....|....|....|....|....|....|....|....|....|....|....|....|....|....|....|....|....|
Homo_sapiens                 TCATCCTCTGCTCCCTCACTTAGGCTCCAATTCCTCCTGATTTTGTTC----TGTTTTTCCCTTCGGGCATCCAAGGCAGCCACCTGCCCAGGCCTGGGC
Pan_troglodytes              TCATCCTCTGCTCCCTCACTTAGGCTCCAATTCCTCCTGATTTTGTTC----TGTTTTTCCCTTCGGGCATCCAAGGCAGCCACCTGCCCAGGCCTGGGC
Pan_paniscus                 TCATCCTCTGCTCCCTCACTTAGGCTCCAATTCCTCCTGATTTTGTTC----TGTTTTTCCCTTCGGGCATCCAAGGCAGCCACCTGCCCAGGCCTGGGC
Gorilla_gorilla              TCATCCTCTGCTCCCTCACTTAGGCTCCAATTCCTCCTGATTTTGTTC----TGTTTTTCCCTTCGGGCATCCAAGGCAGCCACCTGCCCAGGCCTGGGC
Pongo_abelii                 TCATCC-CCACTCCCTCACTTAGGCTCCAATTCCTCCTGATTTTGTTC----TGTTTTTCCCTTTGGGCATCCAAGGCAGCCACCTGCCCAGGCCTGGGC
Nomascus_leucogenys          TCATCCCCTGCTCCCTCACTTAGGCTCCAATTCCTCCTGATTTTGTTC----TGTTTTTCCCTTCGGGTATCCAAGGCAGCCACCTGCCCAGGCCTGGGC
Macaca_nemestrina            CCATCCCCTGCTCCCTCACTTAGGCTCTAATTCCTCCTGATTTCGTTC----TGCTTTTCCCTTCCTGCATCCAAGGCAGACACCTGCCCAGGCCTGGGC
Macaca_fascicularis          CCATCCCCTGCTCCCTCACTTAGGCTCTAATTCCTCCTGATTTCGTTC----TGCTTTTCCCTTCCTGCATCCAAGGCAGATACCTGCCCAGGCCTGGGC
Macaca_mulatta               CCATCCCCTGCTCCCTCACTTAGGCTCTAATTCCTCCTGATTTCGTTC----TGCTTTTCCCTTCCTGCATCCAAGGCAGATACCTGCCCAGGCCTGGGC
Papio_anubis                 CCATCCCCTGCTCCCTCACTTAGGCTCTAATTCCTCCTGATTTCGTTC----TGCTTTTCCCTTCCGGCATCCAAGGCAGACACCTGCCCAGGCCTGGGC
Rhinopithecus_roxellana      TCATCCCCTGCTCCCTCACTTAGGCTCTAATTCCTCCTGATTTTGTTC----TGCTTTTCCCTTCCGGCATCCAAGGCAGACACCTGCCCAGGCCTGGGC
Theropithecus_gelada         CCATCCCCTGCTCCCTCACTTAGGCTCTAATTCCTCCTGATTTCGTTC----TGCTTTTCCCTTCCGGCATCCAAGGCAGACACCTGCCCAGGCCTGGGC
Colobus_angolensis           TCATCCCCTGCTCCCTCACTTAGGCTCTAATTCCTCCTGATTTTGTTC----TGCTTTTCCCTTCCGGCATCCAAGGCAGACACCTGCCCAGGCCTGGGC
Rhinopithecus_bieti          TCATCCCCTGCTCCCTCACTTAGGCTCTAATTCCTCCTGATTTTGTTC----TGCTTTTCCCTTCCGGCATCCAAGGCAGACACCTGCCCAGGCCTGGGC
Piliocolobus_tephrosceles    TCATCCCCTGCTCCCTCACTTAGGCTCTAATTCCTCCTGATTTTGTTC----TGCTTTTCCCTTCCGGCATCCAAGGCAGACACCTGCCCAGGTCTGGGC
Chlorocebus_sabaeus          CCATCCCCTGCTCCCTCACTTAGGCTCTAATTCCTCCTGATTTCGTTC----TGCTTTTCCCTTCCGGCATCCAAGGCAGACACCTGCCCCGGCCTGGGC
Mandrillus_leucophaeus       CCATCCCCTGCTCCCTCACTTAGGCTCTAATTCCTCCTGATTTCGTTC----TGCTTTTCCCTTCGGGCATCCAAGGCAGACACCTGCCCAGGCCTGGGC
Aotus_nancymaae              TCATCTCCTGCTTCCTCACATGGGGTCCAATTCCTCCTGATTTTGTTCTCTGATGTTCTCCCTTC-AGCATCTAGGGCGGCCACCTGCCCAGGCCTGGGT
Callithrix_jacchus           TCATCTCCTGCTTCCTCACATGGGCTCCAATTCCTCCTGATTTTGTTT----TGCTTTTCCCTTCTG-CATCTGGGGCGGCCACCTGCCCAGGCCTGGGC
Cebus_capucinus_imitator     TCATCTCCTGCTTCCTCACGTGGTCTCCAGTTCC------------------TACTTTTCCCTTC-GGCATCTAGGGTGGCCACCTGCCCAGGCCTGTGC
Saimiri_boliviensis          TCATCCCCTGCTTCCTCACATGGGCTCCAGTTC----TGATTTTGTTC----TACTTTTCCCTTC-GGCATCTAGGGTGGTCACCTGCCCAGGCCTGGGC
```

```
                                     610       620       630       640       650       660       670       680       690       700
                           ....|....|....|....|....|....|....|....|....|....|....|....|....|....|....|....|....|....|....|....|
Homo_sapiens               CTGTTCAAACACTGTCTCCGCTGTACTCCC---TAGCTCCTT----GAACC-----CCTACATTGTCTGCATTCAGGAAGTTTTGTGGCTTTTT-AGGATTT
Pan_troglodytes            CTGTTCAAACACTGTCTCCGCTGTACTCCC---TAGCTCCTT----GAACC-----CCTACATTGTCTGCATTCAGGAAGTTTTGTGGCTTTTT-AGGATGT
Pan_paniscus               CTGTTCAAACACTGTCTCCGCTGTACTCCC---TAGCTCCTT----GAACC-----CCTACATTGTCTGCATTCAGGAAGTTTTGTGGCTTTTT-AGGATGT
Gorilla_gorilla            CTGTTCAAACACTGTCTCCGCTGTACTCCC---TAGCTCCTT----GAACC-----CCTACATTGTCTGCATTCAGGAAGTTTTGTGGCTTTTC-AGGATGT
Pongo_abelii               CTGTTCAAACACTGTCTCCGCTGTACTCCC---TAGCTCCTTCCTTGAACC-----CCTACATTGTCTGCATTCAGGAAGTTTTGTGGCTTTTT-AGGATGT
Nomascus_leucogenys        CTGTTCAAACACTGTCTCCGCTGTACTCCC---TGGCTCCTTCCTTGAACC-----CCTACATTGTCTGCATTCAGGAAGTTTTGTGGCTTTTT-AGGATGT
Macaca_nemestrina          CTGCTCAAATACTGTCTCCACTGTACTCCC---TAGCTCCTTCCTTGAACC-----TCTACATTGTCTGCATTCAGGAAGTTTTGTGGCTCTTT-AGGATGT
Macaca_fascicularis        CTGCTCAAATACTGTCTCCACTGTACTCCC---TAGCTCCTTCCTTGAACC-----TCTACATTGTCTGCATTCAGGAAGTTTTGTGGCTCTTT-AGGATGT
Macaca_mulatta             CTGCTCAAATACTGTCTCCACTGTACTCCC---TAGCTCCTTCCTTGAACC-----TCTACATTGTCTGCATTCAGGAAGTTTTGTGGCTCTTT-AGGATGT
Papio_anubis               CTGCTCAAATACTGTCTCCACTGTACTCCC---TAGCTCCTTCCTTGAACC-----TCTACATTGTCTGCATTCAGGAAGTTTTGTGGCTCTTT-AGGATGT
Rhinopithecus_roxellana    CTGTTCAAATACT--------------CCC---TAGCTCCTTCCTTGAACC-----TCTACATTGTCTGCATTCAGAAAGTTTTGTGGCTCTTT-AGGATGT
Theropithecus_gelada       CTGCTCAAATACTGTCTCCACTGTACTCCC---TAGCTCCTTCCTTGAACC-----TCTACATTGTCTGCATTCAGGAAGTTTTGTGGCTCTTT-AGGATGT
Colobus_angolensis         CTGTTCAAATACTGTCTCCACTGTACTCCC---TAGCTCCTTCCTTGAACC-----TCTACATTGTCTGCATTCAGGAAGTTTTGTGACTCTTT-AGGATGT
Rhinopithecus_bieti        CTGTTCAAATACT--------------CCC---TGGCTCCTTCCTTGAACC-----TCTACATTGTCTGCATTCAGAAAGTTTTGTGGCTCTTT-AGGATGT
Piliocolobus_tephrosceles  CTGTTCAAATACTGCCTCCACTGTACTCCCCCTAGCTCCTTCCTTGAACC-----TCTACATTGTCTGCATTCAGGAAGTTTTGTGGCTCTTT-AGGATGT
Chlorocebus_sabaeus        CTGTTCAAATACTGTCTTCGCTGTCCTCCC---TAGCTCCTTCCTTGAACC-----TCTACATTGTCTGCATTCAGGAAGTTTTGTGGCTCTTT-AGGATGT
Mandrillus_leucophaeus     CTGCTCAAATACTGTCTCCACTGTACCCCC---TAGCTCCTTCCTTGAACCGAACCTCTGCATTGTCTGCATTCAGGAAGTTTTGTGGCTCTTT-AGGATGT
Aotus_nancymaae            CTTTCCGAACACTGCTCCCGCTGTACTCCC---CATTTCCTTCTTT----------CCTACAATGTCTGCGTCCAGGAAGTTTTGTGGCTTTTT-AATTTCA
Callithrix_jacchus         CTTTCCGATCACTGCCTCCGCTGTACTCCC----ATCTCCTTCCTTGAACC-----CCTACTATGTCTGCGTCCAGGAAGTTTTGTGGCTTTTT-TATTTCA
Cebus_capucinus_imitator   CTTTCCGAACACTGCCTCCGCTGTACTCCC---CATCTCCTTCCTTGAACC------CTACAACGTCTGCGTCCAGGAAGTTTTGTGGC--TTT--ATTTCA
Saimiri_boliviensis        CTTACTGAACACTGCCTCCGCTGTACTCCC---CATCTCCTTCCTTGAACC------CTACAATGTTTGCGTCCAGGAAGTTTTGCGGCTTTTAAAATTTCA

                                     710       720       730       740       750       760       770       780       790       800
                           ....|....|....|....|....|....|....|....|....|....|....|....|....|....|....|....|....|....|....|
Homo_sapiens               TTTT------------------------------------------------------------------------------------------------
Pan_troglodytes            TTTTT-----------------------------------------------------------------------------------------------
Pan_paniscus               TTTTT-----------------------------------------------------------------------------------------------
Gorilla_gorilla            TTTTTT----------------------------------------------------------------------------------------------
Pongo_abelii               TTTTT-----------------------------------------------------------------------------------------------
Nomascus_leucogenys        TTTTT-----------------------------------------------------------------------------------------------
Macaca_nemestrina          TTATTT----------------------------------------------------------------------------------------------
Macaca_fascicularis        TTATTT----------------------------------------------------------------------------------------------
Macaca_mulatta             TTATTT----------------------------------------------------------------------------------------------
Papio_anubis               TTAGTT----------------------------------------------------------------------------------------------
Rhinopithecus_roxellana    TTTTT-----------------------------------------------------------------------------------------------
Theropithecus_gelada       TTATTT----------------------------------------------------------------------------------------------
Colobus_angolensis         TTTT------------------------------------------------------------------------------------------------
Rhinopithecus_bieti        TTTTT-----------------------------------------------------------------------------------------------
Piliocolobus_tephrosceles  TTTT------------------------------------------------------------------------------------------------
Chlorocebus_sabaeus        TTTT------------------------------------------------------------------------------------------------
Mandrillus_leucophaeus     TTATTT----------------------------------------------------------------------------------------------
Aotus_nancymaae            TTTATTTATTTGTTTATTTATTTATTTATTTGAGATGGAGGCTTGCTGCCTCAGCCTCCTGAGTAGCTGGGA-TACA-TGCCTGGCAGCTGGGATAACAT
Callithrix_jacchus         TTTATTTATTTACTTATTTATTTA----TTTGAGATGGAGGCTTGTTCCCTCACCCTCCCGAGTAGCTGGG-TTACAGGTGCCCTCAACCACGCCCGGCA
Cebus_capucinus_imitator   TTTATTTATTTATTTATTTA--------TTTGAGATGGAGGCTTTCTCCCTCACCCTCCTGAGTAGCTGG-ATTACAGGTGCCCGCCACCACGCTTGGCA
Saimiri_boliviensis        TTTATTTATTTATTTATTCATTTA----TTTGAGATGGAGGCTTGCTCCCTCACACTCCTGAGTAGCTGGGATTACAGTTCCCGGC-ACCACTCCCGGCA
```

```
                                   810       820       830       840       850       860       870       880       890       900
                               ....|....|....|....|....|....|....|....|....|....|....|....|....|....|....|....|....|....|....|....|
Homo_sapiens                   ----------------------------------------------------------------------------------------------------
Pan_troglodytes                ----------------------------------------------------------------------------------------------------
Pan_paniscus                   ----------------------------------------------------------------------------------------------------
Gorilla_gorilla                ----------------------------------------------------------------------------------------------------
Pongo_abelii                   ----------------------------------------------------------------------------------------------------
Nomascus_leucogenys            ----------------------------------------------------------------------------------------------------
Macaca_nemestrina              ----------------------------------------------------------------------------------------------------
Macaca_fascicularis            ----------------------------------------------------------------------------------------------------
Macaca_mulatta                 ----------------------------------------------------------------------------------------------------
Papio_anubis                   ----------------------------------------------------------------------------------------------------
Rhinopithecus_roxellana        ----------------------------------------------------------------------------------------------------
Theropithecus_gelada           ----------------------------------------------------------------------------------------------------
Colobus_angolensis             ----------------------------------------------------------------------------------------------------
Rhinopithecus_bieti            ----------------------------------------------------------------------------------------------------
Piliocolobus_tephrosceles      ----------------------------------------------------------------------------------------------------
Chlorocebus_sabaeus            ----------------------------------------------------------------------------------------------------
Mandrillus_leucophaeus         ----------------------------------------------------------------------------------------------------
Aotus_nancymaae                ---------------------------GCCTGGCTAATTTT-GTATTTTTAGTAGAGACGGAGCTTCTCCATGTTGGTCAGGCTGGTCTTGAACTCC---
Callithrix_jacchus             GCTGGGATTACAGGTGCCCTCAACCACGCCCGGCTAATTTTTGTATTTTTAGAAGACACAGGGTTTCACCATGTTGGCCAGGCTGGTCTTGAACTCCTCA
Cebus_capucinus_imitator       GCTGGGATTACAGGTGCCCGCCACCACGCTTGGCTAATTTTTGTATTTTTAGAAGACACAGGGTTTCACCATGTTGGCCAGGCTGGTCTTGAACCCCTCA
Saimiri_boliviensis            GCTGGGATTACAGGTTCCCGGCACCACTCCCGGCTAATTTTTGTATTTTTAGAAGACACAGAGTTTCGCTATGTTGGCCAGGCTAGTCTTGAACTCCTCA

                                   910       920       930       940       950       960       970       980       990      1000
                               ....|....|....|....|....|....|....|....|....|....|....|....|....|....|....|....|....|....|....|....|
Homo_sapiens                   ----------------------------------------------------------------------------------------------------
Pan_troglodytes                ----------------------------------------------------------------------------------------------------
Pan_paniscus                   ----------------------------------------------------------------------------------------------------
Gorilla_gorilla                ----------------------------------------------------------------------------------------------------
Pongo_abelii                   ----------------------------------------------------------------------------------------------------
Nomascus_leucogenys            ----------------------------------------------------------------------------------------------------
Macaca_nemestrina              ----------------------------------------------------------------------------------------------------
Macaca_fascicularis            ----------------------------------------------------------------------------------------------------
Macaca_mulatta                 ----------------------------------------------------------------------------------------------------
Papio_anubis                   ----------------------------------------------------------------------------------------------------
Rhinopithecus_roxellana        ----------------------------------------------------------------------------------------------------
Theropithecus_gelada           ----------------------------------------------------------------------------------------------------
Colobus_angolensis             ----------------------------------------------------------------------------------------------------
Rhinopithecus_bieti            ----------------------------------------------------------------------------------------------------
Piliocolobus_tephrosceles      ----------------------------------------------------------------------------------------------------
Chlorocebus_sabaeus            ----------------------------------------------------------------------------------------------------
Mandrillus_leucophaeus         ----------------------------------------------------------------------------------------------------
Aotus_nancymaae                ----------------------------------------------------------------------------------------------------
Callithrix_jacchus             C-CTCGTGATCCACCCGCCTTGGCCTCCCAAAGTGTAGGGATTACAGGCATGAGCCACNNNNNNNNNNNNNNNNNNNNNNNNNNNNNNNNNNNNNNNNNN
Cebus_capucinus_imitator       C-CTGGTGATCTACCAGCCTTGGCCTCCCAAAGTGTAGGGATTACAGGCGTGAGCCACCGCGCCCAGCCGGCTTTTTTATTTTTTGAGAGGGAGTTTCAT
Saimiri_boliviensis            C-CTCGTGATCCACCCGCCTTGGCCTCCCAAAGTGTAGGGATTACAGGCTTGAGCCACCGCGCCCAGCCGGCTTTTTTATTTTTTGAGAGGGAGTTTCAC
```

```
                                       1010      1020      1030      1040      1050      1060      1070      1080      1090      1100
                              ....|....|....|....|....|....|....|....|....|....|....|....|....|....|....|....|....|....|....|....|
Homo_sapiens                  ----------------------------------------------------------------------------------------------------
Pan_troglodytes               ----------------------------------------------------------------------------------------------------
Pan_paniscus                  ----------------------------------------------------------------------------------------------------
Gorilla_gorilla               ----------------------------------------------------------------------------------------------------
Pongo_abelii                  ----------------------------------------------------------------------------------------------------
Nomascus_leucogenys           ----------------------------------------------------------------------------------------------------
Macaca_nemestrina             ----------------------------------------------------------------------------------------------------
Macaca_fascicularis           ----------------------------------------------------------------------------------------------------
Macaca_mulatta                ----------------------------------------------------------------------------------------------------
Papio_anubis                  ----------------------------------------------------------------------------------------------------
Rhinopithecus_roxellana       ----------------------------------------------------------------------------------------------------
Theropithecus_gelada          ----------------------------------------------------------------------------------------------------
Colobus_angolensis            ----------------------------------------------------------------------------------------------------
Rhinopithecus_bieti           ----------------------------------------------------------------------------------------------------
Piliocolobus_tephrosceles     ----------------------------------------------------------------------------------------------------
Chlorocebus_sabaeus           ----------------------------------------------------------------------------------------------------
Mandrillus_leucophaeus        ----------------------------------------------------------------------------------------------------
Aotus_nancymaae               ----------------------------------------------------------------------------------------------------
Callithrix_jacchus            NNNNNNNNNNNNNNNNNNNNNNNNNNNNNNNNNNNNNNNNNNNNNNNNNNNNNNNNNNNNNNNNNNNNNNNNNNNNNNNNNNNNNNNNNNNNNNNNNNNN
Cebus_capucinus_imitator      TCTTGTTGCCCAGGCTGGGGTGAAATGGCACAATCTCGGCTCACTGCAACCTCTGCCTCCCCAGTTCAAGCGATTCTCCTGCCTCAGCCTCCTGAGTAGC
Saimiri_boliviensis           TCTTGTTGCCCAGGCTCGAGTGAAATGGCACAATCTCGGCTCACTGCAACCTCTGCCTCCCCAGTTCAAGCGATTCTCCTGCCGCAGCCTCCTGAGTAGC


                                       1110      1120      1130      1140      1150      1160      1170      1180      1190      1200
                              ....|....|....|....|....|....|....|....|....|....|....|....|....|....|....|....|....|....|....|....|
Homo_sapiens                  ----------------------------------------------------------------------------------------------------
Pan_troglodytes               ----------------------------------------------------------------------------------------------------
Pan_paniscus                  ----------------------------------------------------------------------------------------------------
Gorilla_gorilla               ----------------------------------------------------------------------------------------------------
Pongo_abelii                  ----------------------------------------------------------------------------------------------------
Nomascus_leucogenys           ----------------------------------------------------------------------------------------------------
Macaca_nemestrina             ----------------------------------------------------------------------------------------------------
Macaca_fascicularis           ----------------------------------------------------------------------------------------------------
Macaca_mulatta                ----------------------------------------------------------------------------------------------------
Papio_anubis                  ----------------------------------------------------------------------------------------------------
Rhinopithecus_roxellana       ----------------------------------------------------------------------------------------------------
Theropithecus_gelada          ----------------------------------------------------------------------------------------------------
Colobus_angolensis            ----------------------------------------------------------------------------------------------------
Rhinopithecus_bieti           ----------------------------------------------------------------------------------------------------
Piliocolobus_tephrosceles     ----------------------------------------------------------------------------------------------------
Chlorocebus_sabaeus           ----------------------------------------------------------------------------------------------------
Mandrillus_leucophaeus        ----------------------------------------------------------------------------------------------------
Aotus_nancymaae               --------------------------------------------------------------------------------CACCTCAGGTGATCTGCCCG
Callithrix_jacchus            NNNNNNNNNNNNNNNNNNNNNNNNNNNNNNNNNNNNNNNNNNNNNNNNNNNNNNNNNNNNNNNNNNNNNNNNNNNNNNNNNNNNNNNNNNNNNNNNNNNN
Cebus_capucinus_imitator      TGGGATTACGCGCCTGGCTAATTTTGTATTTTTAGTAGAGATGGAGCTTCTCCATGTTCGTCAGGCTGGTCTTGAACTCTCACCTCAGGTGATCTGCCCA
Saimiri_boliviensis           TGGGATTACACGCCTGGCCAATTTTGTATTTTTAGTAGAGACGGAGCTTCTCCATGTTGGTCAGGCTGGTCTTGAACTGCGACCTCAGGTGATCTGCCCA
```

```
                                         1210      1220      1230      1240      1250      1260      1270      1280      1290      1300
                                 ....|....|....|....|....|....|....|....|....|....|....|....|....|....|....|....|....|....|....|....|
Homo_sapiens                     ----------------------------------------------------------------------------------------------------
Pan_troglodytes                  ----------------------------------------------------------------------------------------------------
Pan_paniscus                     ----------------------------------------------------------------------------------------------------
Gorilla_gorilla                  ----------------------------------------------------------------------------------------------------
Pongo_abelii                     ----------------------------------------------------------------------------------------------------
Nomascus_leucogenys              ----------------------------------------------------------------------------------------------------
Macaca_nemestrina                ----------------------------------------------------------------------------------------------------
Macaca_fascicularis              ----------------------------------------------------------------------------------------------------
Macaca_mulatta                   ----------------------------------------------------------------------------------------------------
Papio_anubis                     ----------------------------------------------------------------------------------------------------
Rhinopithecus_roxellana          ----------------------------------------------------------------------------------------------------
Theropithecus_gelada             ----------------------------------------------------------------------------------------------------
Colobus_angolensis               ----------------------------------------------------------------------------------------------------
Rhinopithecus_bieti              ----------------------------------------------------------------------------------------------------
Piliocolobus_tephrosceles        ----------------------------------------------------------------------------------------------------
Chlorocebus_sabaeus              ----------------------------------------------------------------------------------------------------
Mandrillus_leucophaeus           ----------------------------------------------------------------------------------------------------
Aotus_nancymaae                  CCTCGGCCTCCAAAAGTGCTGGGATTATAGGCTCTGCGCCCACCCTTGGCTCCATTTTAGGAAATGTTGTGACTTTTCAGGATTTGTTTTTAAAACACAG
Callithrix_jacchus               NNNNNNNNNNNNNNNNNNNNNNNNNNNNNNNNNNNNNNNNNNNNNNNNNNNNNNNNNNNNNNNNNNNNNNNNNNNNNNNNNNNNNNNNNNNNNNNNNNNN
Cebus_capucinus_imitator         CCTCGGCCACCAAAAGTGCTGGGATTATAGGCTCTGCGCCCACCCTTGGCTCCATTTTAGGAAATTCTGTGACTTTTCAGGATTTGTTTTTAAAACACAG
Saimiri_boliviensis              CCTCGGACTCCAAAAGTGCTGGGATTATAGGCTCTGCGCCCACCCTTGGCTCCATTTTAGGAAATTTTGTGACTTTTCAGGATTTGTTTTTAAAACACAG

                                         1310      1320      1330      1340      1350      1360      1370      1380      1390      1400
                                 ....|....|....|....|....|....|....|....|....|....|....|....|....|....|....|....|....|....|....|
Homo_sapiens                     ----------------------------------------------------------------------------------------------------
Pan_troglodytes                  ----------------------------------------------------------------------------------------------------
Pan_paniscus                     ----------------------------------------------------------------------------------------------------
Gorilla_gorilla                  ----------------------------------------------------------------------------------------------------
Pongo_abelii                     ----------------------------------------------------------------------------------------------------
Nomascus_leucogenys              ----------------------------------------------------------------------------------------------------
Macaca_nemestrina                ----------------------------------------------------------------------------------------------------
Macaca_fascicularis              ----------------------------------------------------------------------------------------------------
Macaca_mulatta                   ----------------------------------------------------------------------------------------------------
Papio_anubis                     ----------------------------------------------------------------------------------------------------
Rhinopithecus_roxellana          ----------------------------------------------------------------------------------------------------
Theropithecus_gelada             ----------------------------------------------------------------------------------------------------
Colobus_angolensis               ----------------------------------------------------------------------------------------------------
Rhinopithecus_bieti              ----------------------------------------------------------------------------------------------------
Piliocolobus_tephrosceles        ----------------------------------------------------------------------------------------------------
Chlorocebus_sabaeus              ----------------------------------------------------------------------------------------------------
Mandrillus_leucophaeus           ----------------------------------------------------------------------------------------------------
Aotus_nancymaae                  GGTTGCCAGGCGCGGTGGCTCACGCCTGGAG---------------------------------------------------------------------
Callithrix_jacchus               NNNNGCCAGGCGCAGTGGCTCACTCCTGTAATCCCAGCACTTTGGGAAGCCAAGGCAGGTGTCTCATGAGGTCAAGAGATGGAGACCATCCTGGCCAACA
Cebus_capucinus_imitator         GGTTGCCAGGCGCAGTGGCTCACGCCTGTAATCCCAGCTCTTTGGGAAGCCAAGGCAGGTGGCTCATGAGGTCAAGAGATGGAGACCATCCTGGCCAACA
Saimiri_boliviensis              GGTTGCCAGGCACAGTGGCTCACGCCTGTAATCCCAGCACTTTGGAAAGTCAAGGCAGGTGGCTCATGAGGTCAAGAAATGGAGACCGTCCTGGCCAACA
```

```
                                         1410      1420      1430      1440      1450      1460      1470      1480      1490      1500
                                 ....|....|....|....|....|....|....|....|....|....|....|....|....|....|....|....|....|....|....|....|
Homo_sapiens                     ----------------------------------------------------------------------------------------------------
Pan_troglodytes                  ----------------------------------------------------------------------------------------------------
Pan_paniscus                     ----------------------------------------------------------------------------------------------------
Gorilla_gorilla                  ----------------------------------------------------------------------------------------------------
Pongo_abelii                     ----------------------------------------------------------------------------------------------------
Nomascus_leucogenys              ----------------------------------------------------------------------------------------------------
Macaca_nemestrina                ----------------------------------------------------------------------------------------------------
Macaca_fascicularis              ----------------------------------------------------------------------------------------------------
Macaca_mulatta                   ----------------------------------------------------------------------------------------------------
Papio_anubis                     ----------------------------------------------------------------------------------------------------
Rhinopithecus_roxellana          ----------------------------------------------------------------------------------------------------
Theropithecus_gelada             ----------------------------------------------------------------------------------------------------
Colobus_angolensis               ----------------------------------------------------------------------------------------------------
Rhinopithecus_bieti              ----------------------------------------------------------------------------------------------------
Piliocolobus_tephrosceles        ----------------------------------------------------------------------------------------------------
Chlorocebus_sabaeus              ----------------------------------------------------------------------------------------------------
Mandrillus_leucophaeus           ----------------------------------------------------------------------------------------------------
Aotus_nancymaae                  ----------------------------------------------------------------------------------------------------
Callithrix_jacchus               TGGTGAAACCCCATCTCTACTAAAAATACAAAAAAA-TTATC-----------TAGCACATGCCTGTAGTCCCAGCTATTCCAGAGGCTGAGGCAGAAGA
Cebus_capucinus_imitator         TGGTGAAACCCCATCTCTACTAAAAATACAAAAAAAATTATCGAGACACATGGTAGCACATGCCTGTAGTCCCAGCTACTCGGGAGGCTGAGGCAGAAGA
Saimiri_boliviensis              TGGTGAAACCCCATCTCGACTAAAAATCCAAAAAAAATTATCTA--GACATGGTAGCGCATGCCTGTAGTCCCAGCTACTCGGGAGGCTGAGGCAGAAGA
```

```
                                         1510      1520      1530      1540      1550      1560      1570      1580      1590      1600
                                 ....|....|....|....|....|....|....|....|....|....|....|....|....|....|....|....|....|....|....|....|
Homo_sapiens                     ----------------------------------------------------------------------------------------------------
Pan_troglodytes                  ----------------------------------------------------------------------------------------------------
Pan_paniscus                     ----------------------------------------------------------------------------------------------------
Gorilla_gorilla                  ----------------------------------------------------------------------------------------------------
Pongo_abelii                     ----------------------------------------------------------------------------------------------------
Nomascus_leucogenys              ----------------------------------------------------------------------------------------------------
Macaca_nemestrina                ----------------------------------------------------------------------------------------------------
Macaca_fascicularis              ----------------------------------------------------------------------------------------------------
Macaca_mulatta                   ----------------------------------------------------------------------------------------------------
Papio_anubis                     ----------------------------------------------------------------------------------------------------
Rhinopithecus_roxellana          ----------------------------------------------------------------------------------------------------
Theropithecus_gelada             ----------------------------------------------------------------------------------------------------
Colobus_angolensis               ----------------------------------------------------------------------------------------------------
Rhinopithecus_bieti              ----------------------------------------------------------------------------------------------------
Piliocolobus_tephrosceles        ----------------------------------------------------------------------------------------------------
Chlorocebus_sabaeus              ----------------------------------------------------------------------------------------------------
Mandrillus_leucophaeus           ----------------------------------------------------------------------------------------------------
Aotus_nancymaae                  ----------------------------------------------------------------------------------------------------
Callithrix_jacchus               ATCACTTGAACCCTGGAGGCAGAGGTTGCAGTAAGCTGAGATCTCACCACTGCACTCCAGCCTGGCGACAGAGCGAGACTCCGTCTCTAAATAAATAAAT
Cebus_capucinus_imitator         ATCACTTGAACCCTGGAGTCAGAGGTTGCAGTGAGCTGAGATCACACCACTGCACTTCAGCCTGGTGACAGAGTGAGACTCTGTCTCTAAATAAATAAAT
Saimiri_boliviensis              ATCGCTTGAACCCTGGAGGCAGAGGTTGCAGTAAGCTGAGATCGCACCATTGCACTCCAGCCTGGCGACAGAGTGAGACTCCGTCTCTAAGCAAACAAAT
```

```
                                 1610      1620      1630      1640      1650      1660      1670      1680      1690      1700
                           ....|....|....|....|....|....|....|....|....|....|....|....|....|....|....|....|....|....|....|....|
Homo_sapiens               --------------------TAAACACAGGGTCTGGCTCTGTTGCCCAGGCCGGAGTGCAGTAGTGTGATCATAGCTCACTGTAACCTCGAACTCCT--G
Pan_troglodytes            --------------------TAAACACAGGGTCTGGCTCTGTTGCCCAGGCCGGAGTGCAGTAGTGTGATCATAGCTCACTGTAACTTCGAACTCCT--G
Pan_paniscus               --------------------TAAACACAGGGTCTGGCTCTGTTGCCCAGGCCGGAGTGCAGTAGTGTGATCATAGCTCACTGTAACCTCGAACTCCT--G
Gorilla_gorilla            --------------------TAAACACAGGGTCTGGCTCTGTTGCCCAGGCCGGAGTGCAGTAGTGTGATCATAGCTCACTGTAACCTCGAACTCCT--G
Pongo_abelii               --------------------TAAACACAGGGTCTGGCTCTGTTGCCCAGGCCGGAGTGCAGTAGTGTGATCATAGCTCACTGTAACCTCGAACTCCT--G
Nomascus_leucogenys        --------------------TAAACACAGGGTCTGGCTCTGTTGCCCAGGCCGGAGTGCAGTAGTGTGATCATAGCTCACTGTAACCTCGAACTCCT--G
Macaca_nemestrina          --------------------TTGAGACGGAGTCTCGCTCTGTTGCCCAGGCCGGAGTGCAGTAGTGTGATCGTAGCTCAGTGTAACCTTGAACTCCT--G
Macaca_fascicularis        --------------------TTGAGACGGAGTCTCGCTCTGTTGCCCAGGCCGGAGTGCAGTAGTGTGATCGTAGCTCCGTGTAACCTCGAACTCCT--G
Macaca_mulatta             --------------------TTGAGACGGAGTCTCGCTCTGTTGCCCAGGCCGGAGTGCAGTAGTGTGATCGTAGCTCAGTGTAACCTCGAACTCCT--G
Papio_anubis               --------------------TTGAGACGGAGTCTCGCTCTGTTGCCCAGGCCGGAGTGCAGTAGTGTGATCGTAGCTCAGTGTAACCTCGAACTCCT--G
Rhinopithecus_roxellana    --------------------TAAACACAGGGTCTGGCTCTGTTGCCCAGGCCGGAGTGCAGTAGTGTGATCGTAGCTCACTGTAACCTCGAACTCCT--G
Theropithecus_gelada       --------------------TTGAGACGGAGTCTCGCTCTGTTGCCCAGGCCGGAGTGCAGTAGTGTGATCGTAGCTCAGTGTAACCTCGAACTCCT--G
Colobus_angolensis         --------------------TAAACACAGGGTCTGGCTCTGTTGCCCAGGCCGGAGTGCAGTAGTGTGATCGTAGCTTACTGTAACCTCGAACTCCT--A
Rhinopithecus_bieti        --------------------TAAACACAGGGTCTGGCTCTGTTGCCCAGGCCGGAGTGCAGTAGTGTGATCGTAGCTCACTGTAACCTCGAACTCCTCTT
Piliocolobus_tephrosceles  --------------------TAAACACAGGGTCTGGCTCTGTTGCCCAGGCCGGAGTGCAGTAGTGTGATCGTAGCTTACTGTAACCTCGAACTCCT--G
Chlorocebus_sabaeus        --------------------TAAACACAGGGTCTGGCTCTGTTGCCCAGGCCGGAGTGCAGGAGTGTGATCGTAGCTCACTGTAACCTCGAACTCCT--G
Mandrillus_leucophaeus     --------------------TTGAGACGGAGTCTCGCTCTGTTGCCCAGGCCGGAGTGCAGTAGTGTGATCGTAGCTCAGTGTAACCTCGAACTCCT--G
Aotus_nancymaae            ----------------------------------------------------------------------------------------------------
Callithrix_jacchus         AATTAATTAATTAAAGAAAATAAACACAGGGTCTGGCTCTGTTGCCCAGGCCAGAGTGCAGTAGTGTGATCATAGTTCACTGTAACCTCGAACTCGT--G
Cebus_capucinus_imitator   AAAT-------------AAATAAACACAGGGTCTGGCTCTGTTGCCCAGGCCAGAGTACAGTAGTGTGATCATAGTTCACTGTAACCTCGAACTCCT--G
Saimiri_boliviensis        AAATAAAT---------AAATAAACACAGGGTCTGGCTCTGTTGCCCAGGCCAGAGTACAGTAGTGTGATCATAGTTCACTGTAACCTCGAACTCCT--G

                                 1710      1720      1730      1740
                           ....|....|....|....|....|....|....|....|
Homo_sapiens               GGCTCCAGCCATAGGAAGCTTTTAATAAAACAACCTTTGC
Pan_troglodytes            GGCTCCAGCCATAGGAAGCTTTTAATAAAACAACCTTTGC
Pan_paniscus               GGCTCCAGCCATAGGAAGCTTTTAATAAAACAACCTTTGC
Gorilla_gorilla            GGCTCCAGCCATAGGAAGCTTTTAATAAAACAACCTTTGC
Pongo_abelii               GGCTCCAGCCATAGGAAGCTTTTGATAAAACGACCTTTGC
Nomascus_leucogenys        GGCCCCAGCCATAGGAAGCTTTTGATAAAACAACCCAGGC
Macaca_nemestrina          GGCTCCAACCATAGGAAACCTTTGATAAAACAACCTTTGC
Macaca_fascicularis        GGCTCCAACCATAGGAAACCTTTGATAAAACAACCTTTGC
Macaca_mulatta             GGCTCCAACCATAGGAAACCTTTGATAAAACAACCTTTGC
Papio_anubis               GGCTCCAACCATAGGAAACCTTTGATAAAACAACCTTTGC
Rhinopithecus_roxellana    GGCTCCAACCATAGGAAATCTTTGCTAAAACAACCTTTGC
Theropithecus_gelada       GGCTCCAACCATAGGAAACCTTTGATAAAACAACCTTTGC
Colobus_angolensis         GGCTCCAAATATAGGAAACCTTTGCTAAAACAACCTTTGC
Rhinopithecus_bieti        TGCTCCGACCACACCCTGGCCTTGCTGAATAAAGCTTTGC
Piliocolobus_tephrosceles  GGCTCCAACCATAGGAAACCTTTGCTAAAACAACCTTTGC
Chlorocebus_sabaeus        GGCTCCAACCATAGGAAACCTTTGATAAAACAACCTTTGC
Mandrillus_leucophaeus     GGCTCCAACCATAGGAAACCTTTGATAAAACAACCTTTGC
Aotus_nancymaae            -----TAGCCATAGGAAGCTTTTCATAAACCAACCTTTGC
Callithrix_jacchus         GGCTCTAGCCATAGGAAGCTTTTCATAAGCCAACCTTTGC
Cebus_capucinus_imitator   GGCTCTAGCCATAGGAAGCTTTTCATAAACCAACCTTTGC
Saimiri_boliviensis        GGCTCCAGCCATAGGAAGCTTTTCATAAGCCAACCTTTGC
```

S3 Fig. eNEMAL ortholog alignment. Alignment generated as described in Figure 3. Accession numbers: *Homo sapiens* MT773342, *Pan troglodytes* NC_036890, *Pan paniscus* CM003394, *Gorilla gorilla* NC_044613, *Pongo abelii* NC_036914, *Nomascus leucogenys* NC_044384, *Macaca fascicularis* NC_022285, *Macaca mulatta* NC_041767, *Macaca nemestrina* KQ007745.1, *Papio anubis* NC_044989, *Rhinopithecus bieti* MCGX01000834.1, *Rhinopithecus roxellana* KN295605.1, *Theropithecus gelada* QGDE01000660.1, *Colobus angolensis* KN980607.1, *Piliocolobus tephrosceles* PDMG02000207.1, *Chlorocebus sabaeus* NC_044384, *Mandrillus leucophaeus* KN979072.1, *Aotus nancymaae* KZ200996.1, *Callithrix jacchus* NC_048393, *Cebus capucinus imitator* KV389528.1, and *Saimiri boliviensis* JH378199.1.
