# Supplementary material for: eNEMAL, an enhancer RNA transcribed from a distal MALAT1 enhancer, promotes NEAT1 long isoform expression
Source: PLoS One. 2021 May 21;16(5):e0251515. doi: 10.1371/journal.pone.0251515 (PMC8139514; doi:10.1371/journal.pone.0251515)
Supplement: S4 Fig — The indicated cell lines were exposed to hypoxia for 24 hrs and the fold increases of VEGFA and CA9 were calculated (vs. normoxia). Bar graphs show average ± SD, n = 3. VEGFA and CA9 gene expression levels were normalized to the level of YWHAZ expression. (PDF) [file pone.0251515.s004.pdf]

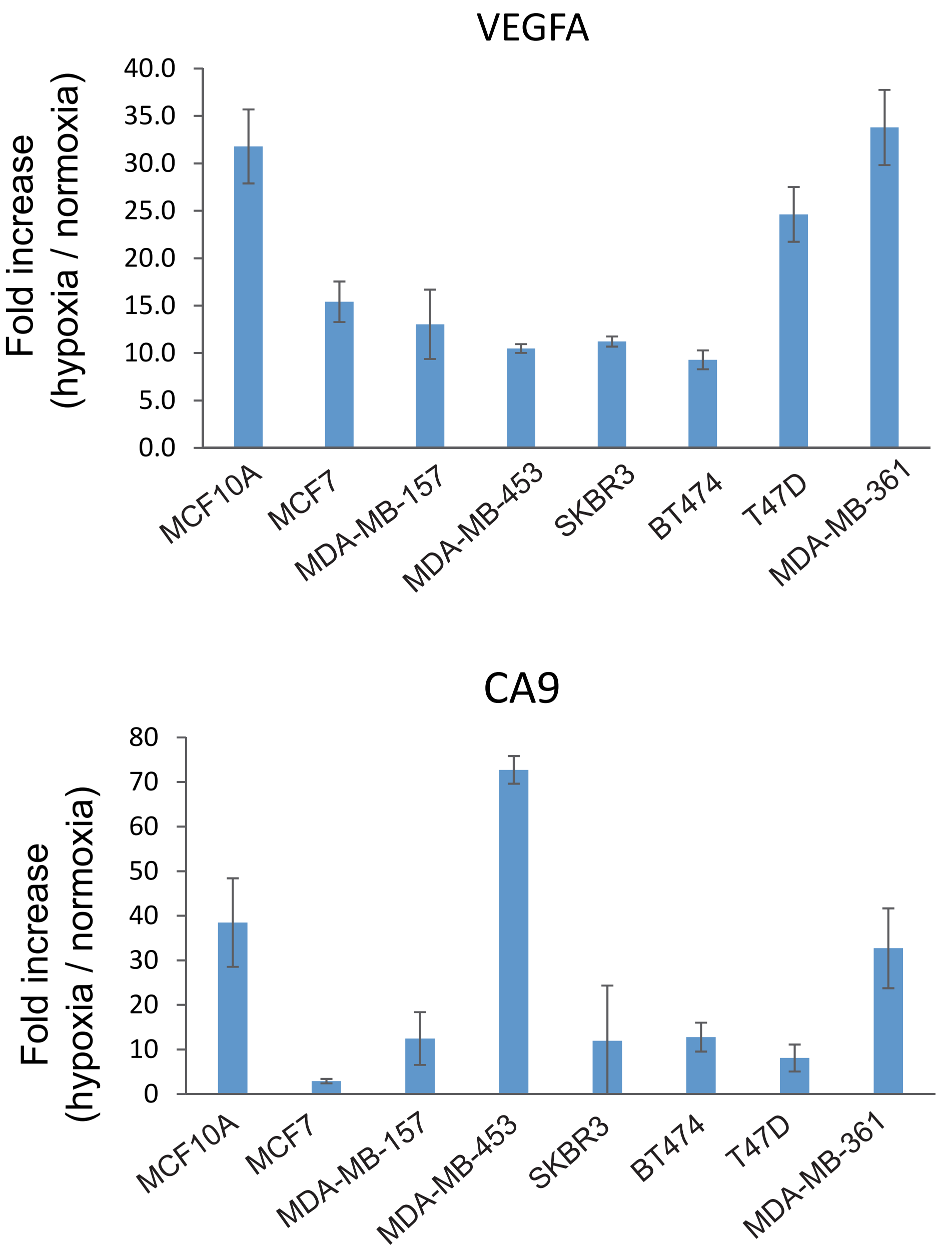

S4 Fig. Upregulation of VEGFA and CA9, well-known hypoxia-induced genes, upon hypoxia. The indicated cell lines were exposed to hypoxia for 24 hrs and the fold increases of VEGFA and CA9 were calculated (vs. normoxia). Bar graphs show average ± SD, n=3. VEGFA and CA9 gene expression levels were normalized to the level of YWHAZ expression.
