# Supplementary material for: eNEMAL, an enhancer RNA transcribed from a distal MALAT1 enhancer, promotes NEAT1 long isoform expression
Source: PLoS One. 2021 May 21;16(5):e0251515. doi: 10.1371/journal.pone.0251515 (PMC8139514; doi:10.1371/journal.pone.0251515)
Supplement: S5 Fig — (A) Fold increases of total NEAT1 and the long isoform (NEAT1_2) upon hypoxic exposure (24 hrs) determined by RT-qPCR using the primer sets indicated in Fig 5D. Note that total NEAT1 and NEAT1_2 are not always proportionally increased, suggesting that hypoxia leads to alternations in the isoform ratio in addition to NEAT1 transcription. (B) Fold increases of NEAT1_2 normalized to fold increases of total NEAT1. Note several breast cancer cell lines with high levels of eNEMAL expression under hypoxia (shown in Fig 4A) show the bigger increase of NEAT1_2 upon hypoxia, suggesting eNEMAL contribution to promoting NEAT1_2 expression. MCF10A, which is a non-tumorigenic line, does not follow the correlation. (PDF) [file pone.0251515.s005.pdf]

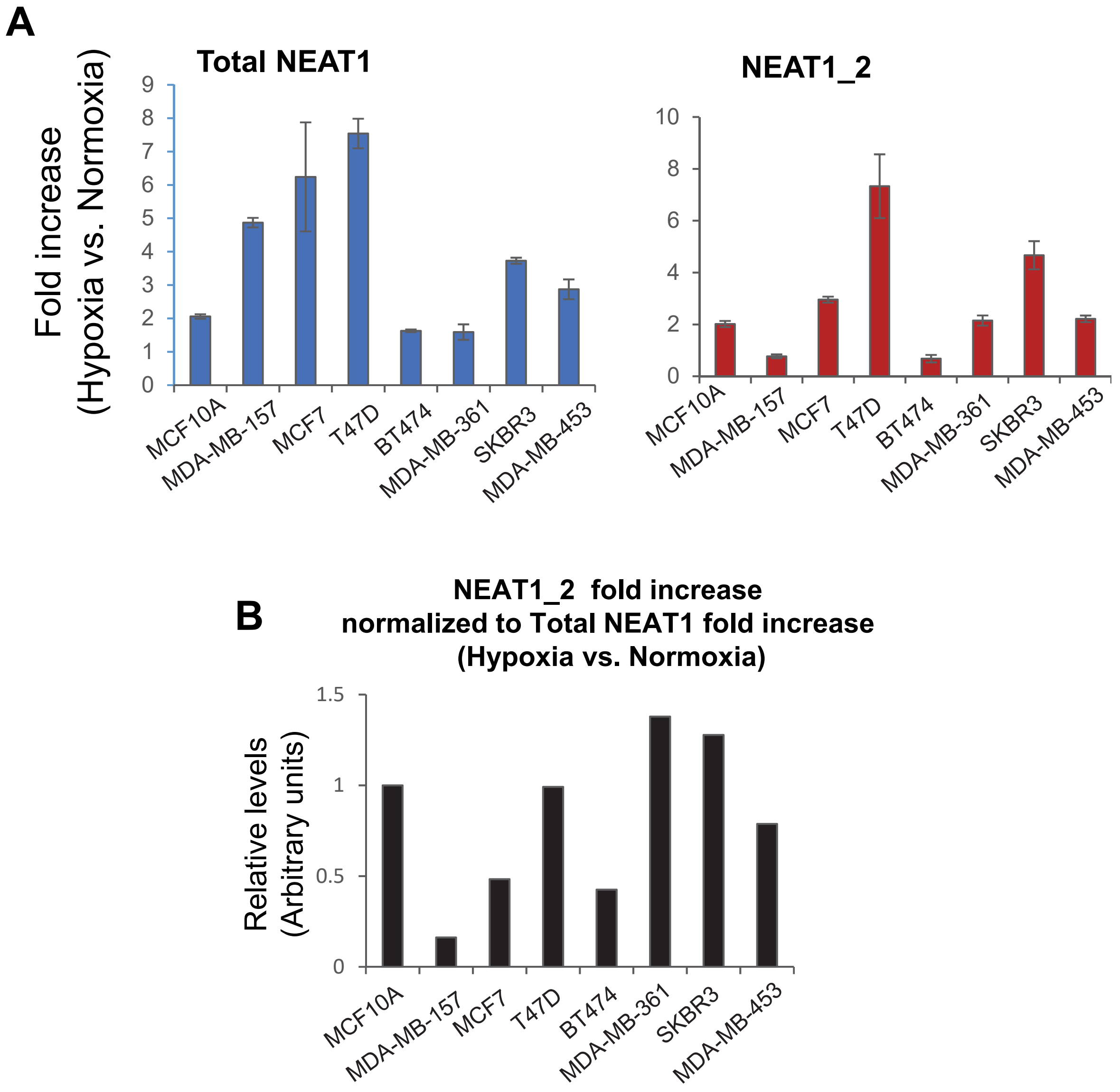

S5 Fig. Total NEAT1 and the NEAT1 long isoform (NEAT1_2) are differentially regulated in different breast cancer cell lines upon hypoxia, and the high levels of NEAT1_2 induction upon hypoxia were observed in some breast cancer cell lines with the high levels of hypoxia-mediated eNEMAL induction.

(**A**) Fold increases of total NEAT1 and the long isoform (NEAT1_2) upon hypoxic exposure (24 hrs) determined by RT-qPCR using the primer sets indicated in Fig 5D. Note that total NEAT1 and NEAT1_2 are not always proportionally increased, suggesting that hypoxia leads to alternations in the isoform ratio in addition to NEAT1 transcription. (**B**) Fold increases of NEAT1_2 normalized to fold increases of total NEAT1. Note several breast cancer cell lines with high levels of eNEMAL expression under hypoxia (shown in Fig 4A) show the bigger increase of NEAT1_2 upon hypoxia, suggesting eNEMAL contribution to promoting NEAT1_2 expression. MCF10A, which is a non-tumorigenic line, does not follow the correlation.
