# Supplementary material for: eNEMAL, an enhancer RNA transcribed from a distal MALAT1 enhancer, promotes NEAT1 long isoform expression
Source: PLoS One. 2021 May 21;16(5):e0251515. doi: 10.1371/journal.pone.0251515 (PMC8139514; doi:10.1371/journal.pone.0251515)
Supplement: S2 Table — (DOCX) [file pone.0251515.s007.docx]

**S2 Table. Primers used in this study.**

| **Purpose** | **Primer Name** | **5'-3' Sequence** |
| --- | --- | --- |
| qPCR (Fig 2A) | -21.3kb_F | CCTAAGCCTTGCTCCAGTTACTG |
| qPCR (Fig 2A) | -21.3kb_R | TCTCCTTAGACTGGGTGTGAGG |
| qPCR (Fig 2A) | -21.0kb_F | AGTTTTGGGAAAGGAGTTCTGG |
| qPCR (Fig 2A) | -21.0kb_R | CACTGTAACCTCGAACTCCTG |
| qPCR (Fig 2A) | -20.9kb_F | GCTGTAACCCAAGAGAGAGGC |
| qPCR (Fig 2A) | -20.9kb_R | AACCCCTACATTGTCTGCATTCAG |
| qPCR (Fig 2A) | -20.7kb_F | AGTACAGCGGAGACAGTGTTTG |
| qPCR (Fig 2A) | -20.7kb_R | TTCTCGCCCTTCATCCTCTG |
| qPCR (Figs 2A, 3A, 3C, 4A, 4B) | -20.5kb_F (=eNEMAL_R) | AGATACCGTTTTCCCGACTTTGG |
| qPCR (Figs 2A, 3A, 3C, 4A, 4B) | -20.5kb_R (=eNEMAL_F) | TCCGCCTTGGGATTTGGG |
| qPCR (Fig 2A) | -20.3kb_F | CTCAAGGCTGGAACAGCG |
| qPCR (Fig 2A) | -20.3kb_R | ACCGCATCGGAGAGAGTC |
| qPCR (Fig 3C) | HIF1A_F | GAACGTCGAAAAGAAAAGTCTCG |
| qPCR (Fig 3C) | HIF1A_R | CCTTATCAAGATGCGAACTCACA |
| qPCR (Fig 3C) | HIF2A_F | GGAGATTCGTGAGAACCTGAGTCTC |
| qPCR (Fig 3C) | HIF2A_R | CATGAAGAAGTCCCGCTCTGTG |
| qPCR (Figs 4A, 4B) | MALAT1_F | GACGGAGGTTGAGATGAAGC |
| qPCR (Fig 4A, 4B) | MALAT1_R | ATTCGGGGCTCTGTAGTCCT |
| qPCR (Figs 4D, 4E) | hNEAT1_1F | GGGAGAGGGTTGGTTAGAGATA |
| qPCR (Figs 4D, 4E) | hNEAT1_1R | TCACCCACGCACTAAATTCC |
| qPCR (Figs 4D, 4E) | hNEAT1_2F | TGGGTTGGTTCCACATCTTT |
| qPCR (Figs 4D, 4E) | hNEAT1_2R | AATCCCACTCCTGGGTATCT |
| qPCR (Figs 2A, 3A, 3C, 4A, 4B, 4D, 4E) | YWHAZ_F | ACTTTTGGTACATTGTGGCTTCAA |
| qPCR (Figs 2A, 3A, 3C, 4A, 4B, 4D, 4E) | YWHAZ_R | CCGCCAGGACAAACCAGTAT |
| RACE (Fig 2B) | 5’RACE_1F | GCATGGGGCTGACAAAGCC |
| RACE (Fig 2B) | 5’RACE_2F | AACGGAGACCACAACACCC |
| RACE (Fig 2B) | Adaptor-oligo(dT) | GCTCGCGAGCGCGTTTAAACGCGC  ACGCGTTTTTTTTTTTTTTTTTTVN |
| RACE (Fig 2B) | Adaptor-targeting 1R | GCTCGCGAGCGCGTTTAAAC |
| RACE (Fig 2B) | Adaptor-targeting 2R | GCGTTTAAACGCGCACGCGT |
| RACE (Fig 2B) | 3’RACE_1F | TCCGCCTTGGGATTTGGG |
| RACE (Fig 2B) | 3’RACE_2F | TTCTCGCCCTTCATCCTCTG |
| RACE (Fig 2B) | Adaptor ligated to RNA | ACGCGTGCGCGTTTAAACGCGCTCGCGAGC |
