# Supplementary material for: eNEMAL, an enhancer RNA transcribed from a distal MALAT1 enhancer, promotes NEAT1 long isoform expression
Source: PLoS One. 2021 May 21;16(5):e0251515. doi: 10.1371/journal.pone.0251515 (PMC8139514; doi:10.1371/journal.pone.0251515)
Supplement: S3 Table — (DOCX) [file pone.0251515.s008.docx]

**S3 Table. siRNA and GapmeR sequences used in this study.**

| **siRNA** | | | |
| --- | --- | --- | --- |
| **Target** | **Sense** | | **Antisense** |
| Negative Control | UAACGACGCGACGACGUAAtt | | UUACGUCGUCGCGUCGUUAUg |
| HIF1A | CAAAGUUCACCUGAGCCUAtt | | UAGGCUCAGGUGAACUUUGtt |
| HIF2A | GCAAAUGUACCCAAUGAUAtt | | UAUCAUUGGGUACAUUUGCtt |
| eNEMAL (siRNA-1) | GACCUAACUUGAGCCUUCAGUtt | | ACUGAAGGCUCAAGUUAGGUCtt |
| eNEMAL (siRNA-2) | GGCUCCAAUUCCUCCUGAUUUtt | | AAAUCAGGAGGAAUUGGAGCCtt |
| Antisense LNA GapmerRs | | | |
| GapmeR Negative Control A (Qiagen cat.no. 339515 LG000000020-DDA) | | A*A*C*A*C*G*T*C*T*A*T*A*C*G*C  (*LNA) | |
| eNEMAL GapmeR (Qiagen cat.no. 339511 LG00778462-DDA – custom designed) | | G*G*A*T*G*C*C*C*G*A*A*G*G*G*A*A  (*LNA) | |
