# Supplementary figures and images for: eNEMAL, an enhancer RNA transcribed from a distal MALAT1 enhancer, promotes NEAT1 long isoform expression
Source: PLoS One. 2021 May 21;16(5):e0251515. doi: 10.1371/journal.pone.0251515 (PMC8139514; doi:10.1371/journal.pone.0251515)

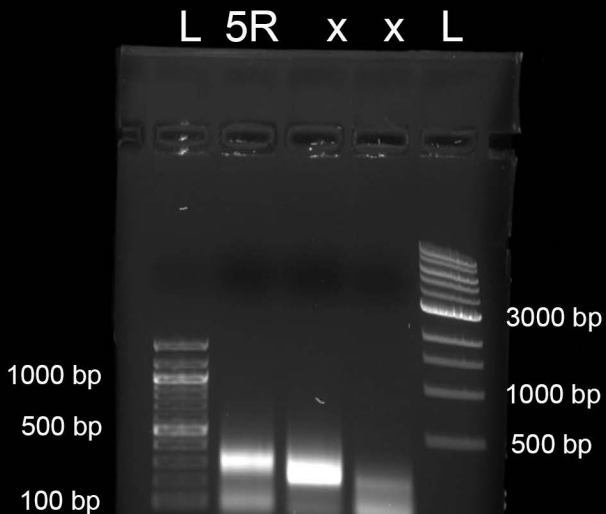

Fig 2C  
5' RACE

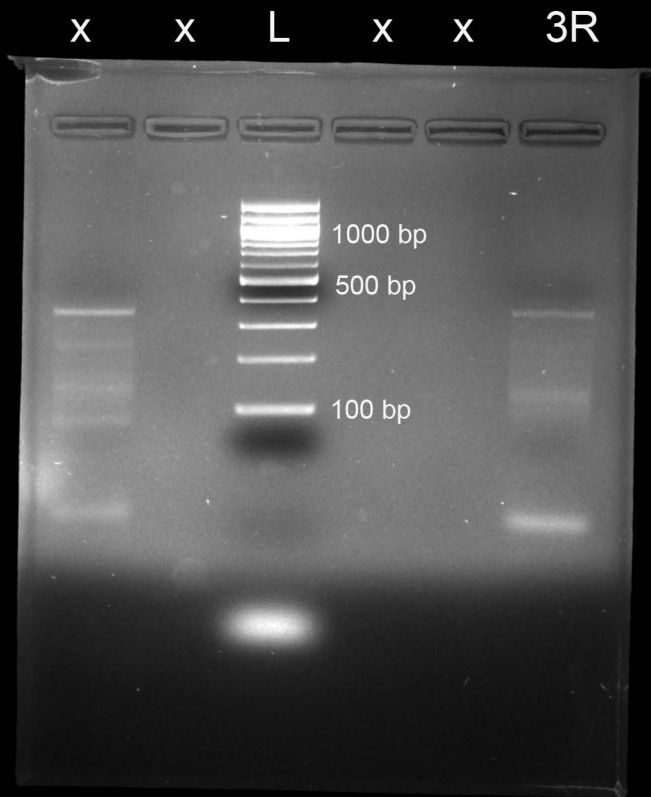

Fig 2C  
S1 Fig  
3' RACE

x x 3R x L x x L x x x x

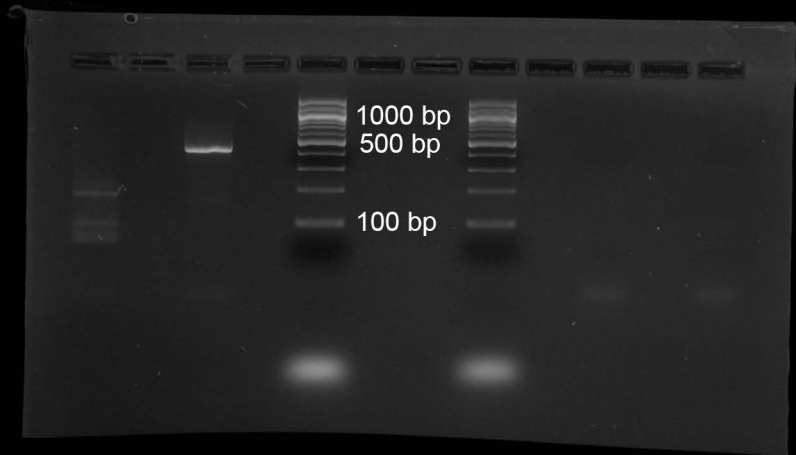

Fig 2C  
S2 Fig  
3' RLM RACE

Supplement: S1 Raw images — (PDF) [file pone.0251515.s009.pdf]
